# People’s Response to Potential Natural Hazard-Triggered Technological Threats after a Sudden-Onset Earthquake in Indonesia

**DOI:** 10.3390/ijerph18073369

**Published:** 2021-03-24

**Authors:** Fatma Lestari, Yasuhito Jibiki, Daisuke Sasaki, Dicky Pelupessy, Agustino Zulys, Fumihiko Imamura

**Affiliations:** 1 Occupational Health & Safety Department, Faculty of Public Health, Universitas Indonesia, Depok, Java Barat 16424, Indonesia; fatma@ui.ac.id or; 2 Disaster Risk Reduction Center, Universitas Indonesia, Depok, West Java 16424, Indonesia; 3 Next Generation Volcano Researcher Development Program, Graduate School of Science, Tohoku University, Sendai, Miyagi 980-8577, Japan; 4 International Research Institute of Disaster Science, Tohoku University, Sendai, Miyagi 980-8577, Japan; dsasaki@irides.tohoku.ac.jp (D.S.); imamura@irides.tohoku.ac.jp (F.I.); 5 Faculty of Psychology, Universitas Indonesia, Depok, West Java 16424, Indonesia; dickypsy@ui.ac.id; 6 Chemistry Department, Faculty of Mathematics and Natural Sciences, Universitas Indonesia, Depok, West Java 16424, Indonesia; zulys@ui.ac.id

**Keywords:** natural hazard-triggered technological (Natech), risk perception, protective actions, evacuation, household survey, Cilegon, Indonesia

## Abstract

(1) Background: We aim to examine whether people activate initial protection behavior, adopt evacuation behavior, worry about the possibility of a tsunami, and consider natural hazard-triggered technological (Natech) situations in a sudden-onset earthquake. The literature suggests that risk perception is a significant predictor of people’s response to potential Natech threats. We aim to empirically verify the variables relating to people’s responses. (2) Methods: We conducted a household survey following a January 2018 earthquake in Indonesia. (3) Results: Immediately after the earthquake, almost 30% of the respondents assembled at the evacuation point. However, sequential steps of people’s response were not observed: evacuation immediately after the earthquake was due to worry about the possibility of a tsunami, but this worry was not related to Natech damage estimation. The relevant factors for evacuation behavior were information access, worry about the possibility of a tsunami, and knowledge of groups and programs related to disaster risk reduction (DRR). The survey location (two villages), perceived earthquake risk, and DRR activity participation are less relevant to the behavior of assembling at the evacuation point. (4) Conclusions: Contrary to the existing literature, our results do not support that higher risk perception is associated with evacuation behavior, or that immediate evacuation is related to foreseeing cascading sequential consequences.

## 1. Introduction

### 1.1. Policy Background on Natech

The description “natural hazard-triggered technological” (Natech) for threats or accidents is an emerging technical term for comparison with familiar natural hazard types. Recently, it has become recognized globally in the context of the Sustainable Development Goals (SDGs) and Sendai Framework for Disaster Risk Reduction (SFDRR). The related SDG target addresses specific aspects of Natech in “3.D National and global health risks,” “11.3 Urbanization,” “12 Consumption and production,” and “13 Climate-related hazards.” Paragraph 15 of the SFDRR notes that its scope includes “related environmental, technological and biological hazards and risks.”

According to an Organisation for Economic Co-operation and Development survey [1] (p. 35, para. 59), there is low visibility of Natech events in risk communication systems. For example, 6 respondents out of 17 samples (14 countries and 3 institutions representing science and industry) stated that information had been “provided to the public in case of emergencies due to chemical accidents” [1] (p. 36). This result indicates that people do not find information provision to be sufficient and this lack of information may not cause preferable behavior for safety in a crisis.

The United Nations Office for Disaster Risk Reduction Asia-Pacific Science Technology and Academia Advisory Group [2] recommends the need for an early warning mechanism, awareness, and training as important activities for Natech risk management. The United Nations Office for Disaster Risk Reduction [3,4] emphasizes almost the same consideration. These policy papers implicitly indicate that warning development, awareness raising, and training opportunities have not been well organized so far. The Izmit Earthquake (also known as the Kocaeli Earthquake) that struck Turkey in 1999, causing a massive fire at the Tupras Izmit refinery and an acrylonitrile spill at the Aksa acrylic fiber production plant, was showcased by Girgin [5] to describe the complexities and great difficulties involved in the evacuation process. More than 20 years have passed since the Izmit Natech incident, and yet still the world has not developed well-prepared methods for people to react to Natech threats.

### 1.2. Case Description of a Potential Natech Threat

Even though policies have not yet been well standardized, in reality, industrial parks are exposed to potential Natech threats. As a case study, we adopt a city in Indonesia, Cilegon city, to analyze how local people respond to Natech threats.

Cilegon is located on the western edge of the Java islands and is known as one of the most well-known and significant heavy industrial areas in Indonesia [6,7,8,9,10,11]. The government of Indonesia and the Association of Southeast Asian Nations (ASEAN) have paid close attention to the potential threats of Natech scenarios in Cilegon. After the Indian Ocean Tsunami of 2004, the Indonesian government conducted a national tsunami simulation for Cilegon in 2007 taking full account of potential Natech threats [12]. In addition, the Cilegon city government developed a tsunami early warning system [13]. These efforts finally culminated in a large-scale preparation exercise in November 2018, the ASEAN Regional Disaster Emergency Response Simulation Exercise (ARDEX 2018), which was conducted in Cilegon [14]. For this reason, Cilegon was selected for our study.

While Jibiki et al. [15] revealed that the community surrounding the industrial facilities in Cilegon was aware of Natech risks, Pelupessy et al. [16] clarified that such awareness was not necessarily connected with organized behaviors in the case of the Anak Krakatau eruption and tsunami, which occurred on the night of Saturday 22 December 2018. Even though no huge tsunami reached the coastal areas in Cilegon in the Anak Krakatau case, great confusion and social disorder were observed [16].

Cilegon experienced an event prior to the Anak Krakatau case. An earthquake of magnitude 6.4 was recorded on Tuesday 23 January 2018, 13:34:50 (local time), and people felt the ground shaking in Cilegon. According to the Indonesian Metrological Agency (Badan Meteorologi Klimatologi dan Geofisika, BMKG), the nearest observation station of our survey area detected “MMI IV (Modified Mercall Intensity)” [17]. This intensity means that the perceived shaking is light and there is no potential damage [17]. However, the local media reported that hundreds of employees felt the shock at one of the major petrochemical factories in Cilegon and evacuated out of the building [18]. The earthquake did not cause a tsunami, and no tsunami warning was issued. We focus on this earthquake in the present study.

Considering the geographical and socioeconomic characteristics of Cilegon, a preferable action in relation to Natech seems to be identified. When shaking is felt in Cilegon, the most preferable action is evacuation to higher grounds after the initial protection behavior (drop, cover, and hold). Since it is difficult to determine whether the epicenters of earthquakes are located inland or are megathrust, it seems preferable to consider the likelihood of tsunamis associated with earthquakes. A warning may be issued, but it is better to save time for evacuation without waiting for a warning. In addition, Natech issues need to be considered. Such a sequential relationship of response is desirable for those living near the industrial park in coastal areas in Cilegon.

### 1.3. Literature Review

While the policy settings have not been well synthesized to achieve the desired evacuation behavior, as noted earlier, we can refer to some literature on the factors generating evacuation behavior specifically for Natech events. Yu et al. [19] used a case study of a fire at a refinery caused by the tsunami triggered by the Great East Japan Earthquake and analyzed factors influencing evacuation behavior. Yu et al. [19] stated that only a few studies [20,21] have examined risk perception of and protective actions against technological threats. According to Yu et al.’s [19] logistic regression analysis to predict households’ immediate evacuation, the first significant predictor is “respondents’ direction to the industrial park” and the second is perceived severity of the Natech threat once they had perceived that a Natech accident would occur. For other factors, Yu et al. [19] stated that households were more likely to evacuate immediately if they felt that their lives or property would be impacted by the Natech accident to a very great extent when they perceived its occurrence. Furthermore, with reference to some studies [20,22,23,24], they pointed out that demographic variables have weak and inconsistent correlations with risk perception and protective responses.

Although Lindell et al. [25] did not deal directly with Natech events, they comprehensively examined the immediate behavioral responses to earthquakes. They concluded that risk perceptions matter for immediate responses to earthquakes, but no previous studies appear to have addressed this matter [25]. Earthquake information and emergency preparedness were associated with lower levels of negative emotions and maladaptive behavior, as well as with increased levels of adaptive behavior; this is one of their most important findings because it supports the effectiveness of pre-impact training activities [25]. Furthermore, they argued that fear was positively related to immediate evacuation. Lindell et al. [25] connected this point to past research and theorized that fear does not necessarily produce loss of control or non-rational flight [26,27,28].

While Yu et al. [19] and Lindell et al. [25] paid attention to psychological aspects, there is also relevant literature in the discipline of safety science. Feng et al. [29] studied post-earthquake evacuation using verbal protocol analysis in immersive virtual reality. The results of their experiments show that participants had wait-or-flight responses in post-earthquake evacuation. They also revealed that people’s decision making tended to be driven, at least partially, by what those around them were doing in the greatest numbers [30]. In addition, Feng et al. [29] found that participant behavior was particularly influenced by those who appeared to be in authority positions, which has been observed in real-life evacuation cases [31,32,33]. Nascimento and Alencar [34] conducted a systematic review of the literature on Natech events, but their study provided few insights on people’s responses. A systematic literature review by Suarez-Paba et al. [35] identified that only 6.1% of the total studies analyzed dealt with risk communication and risk perception. Yu et al. [19] and Yu and Hokugo [36] highlighted the fact that inhabitants’ risk perception triggers their protective behavior (e.g., time to evacuate their house) during a disaster and that this is influenced by such parameters as location, demographic characteristics, and age.

### 1.4. Research Question and Hypothesis

As stated in Section 1.2, we primarily examine the sequential relationship of response as the research question, even though it is quite a hypothetical assumption: the evacuation action is required after the initial protection behavior (drop, cover, and hold). Worry about the possibility of a tsunami is also important, and such emotion needs to be linked with damage estimation, which could be induced by Natech events.

As summarized in Table 1, the existing literature provides relevant factors that seem to influence behavior. We employ proxy variables to verify whether we can obtain similar results to those in the existing literature. Our variables are set from a household-basis questionnaire survey, which we explain in detail in the next section. For the factor of “Direction,” we use “Village location” because almost all the villages in Cilegon are located to the east of the industrial facilities due to the topographical characteristics of Cilegon. As an alternative variable, we test whether the differences in villages may affect behavior (the two villages are coded as binary data). Regarding demographic data, we do not include individuals’ gender and age since we use a household survey in which each respondent provides answers on behalf of the household.

The rest of the paper is structured as follows. Section 2 introduces the survey design and data. Following the Method section, we verify our hypothetical assumption (sequential steps of people’s response) in Section 3.1. In addition, we examine whether risk perception plays a significant role in accordance with earlier works in Section 3.2. In addition to risk perception, other aspects are analyzed to clarify whether they are related to Natech damage estimation (Section 3.3). In contrast to the existing literature, village location, information access, and preparedness are investigated (Section 3.3, Section 3.4, Section 3.5). Section 4 summarizes the results of our analysis, concludes whether we find similar findings to the earlier works, and states the limitations of our study.

## 2. Method

### 2.1. Survey Design

A household survey was carried out at two sites in Cilegon city. The first survey was conducted in Lebak Gede village (“village” here is translation of “kelurahan” in the local language and context) during 14–18 February 2018, while the second survey was conducted in Gunung Sugih village from 6 to 9 March 2018. The sample size was determined in proportion to the total number of households in Lebak Gede (2907 households) and those in Gunung Sugih (1945 households). In order to collect the sufficient number that well represents the total households (4852) of the two villages, we initially planned to gather 500 samples. The samples were proportionally distributed in accordance with the number of households at the neighborhood association level (Rukun Tetangga, or RT) in each village. It was possible to specifically identify the number of households in each RT, and we calculated the composition ratio for each RT. For example, one RT in Gunung Sugih village has 107 households. The composition ratio was calculated as follows: the denominator was 4852 and the numerator was 107. Then, the composition ratio was 0.022052762. We multiplied the composition ratio in the RT by 500 samples (as the expected total number of samples), and we could determine the sample number (11.02638087) in the said RT. Technically, the calculated value was rounded off to the first decimal place, and we finally obtained 11 samples in this example. Finally, 497 samples (299 samples from Lebak Gede and 198 samples from Gunung Sugih) were collected. The enumerators (survey interviewers) were nominated and trained from Indonesian Red Cross volunteers and local residents.

The questionnaire used in this survey was constructed through an elicitation process learned from the interviews (including group interviews) with community leaders and members prior to the survey. Face validity of the questionnaire was acquired through enumerator training in which one session in the training was dedicated to obtaining feedback from the enumerators regarding the readability of the questionnaire items. Enumerator training was conducted to ensure that the enumerators followed the survey protocol and maintained its reliability.

The total number of the question items was 54 in our questionnaire. In the elicitation process for developing our questionnaire, we referred to the relevant literature for each factor. In risk perception, as one of the core factors in our study, we referred to Yu et al. [19] and Lindel et al. [25]. For evacuation behavior, we mainly referred to Yu et al. [19], Lindel et al. [25], and Feng et al. [29]. Insights from Jibiki et al. [15] and Yu et al. [19] were helpful for considering question items regarding Natech damage estimation.

### 2.2. Data Set

In our questionnaire, we included questions asking whether the respondents felt the shaking of the earthquake and whether they stayed in their villages. A total of 380 respondents (76.5% of the total samples; 231 from Lebak Gede, and 149 from Gunung Sugih) answered that they felt the shaking and that they stayed in the village when the earthquake occurred. Based on the chi-square test, we did not find a statistical difference between the two survey locations, and thus there was no need to deal with the data separately. After analyzing the data, we focused on the selected respondents (380 respondents) and those who experienced the earthquake. All respondents did not necessarily answer all questions. In analysis, the total number of the respondents does not reach 380 in some results due to the deficit values.

For reference, we introduced income information relating to our research target locations. Our targeted households in Cilegon city are located in Banten Province. Table 2 shows “Average of net wage/salary per month of formal employee by province and main occupation.” The income level of Banten Province in 2018 was approximately the same as the average level and lower than that of Jakarta Special Province.

For the data analysis, we used IBM SPSS Statistics 22 (IBM Cooperation, New York, the United States). Our study adopted a level of less than 5% to assess the statistical significance of the analysis.

### 2.3. Logistic Regression Analysis

In Section 3.3, we conduct a logistic regression analysis to examine whether some variables may be related to Natech damage estimation. In the analysis, the cases in which respondents answered “I don’t know” are listwise deleted. First, we input the following 16 independent variables: worry about the possibility of a tsunami, evacuation behavior, access to information sources (6 variables), participation in DRR activities, and PMI activities (7 variables). Subsequently, we identify the best model using the stepwise method based on the Akaike information criterion (AIC).

## 3. Results and Interpretations

### 3.1. Sequential Steps of People’s Response

As stated in Section 1.4, the primary purpose of this study is to verify whether sequential steps of the response can be observed. First, 29.2% (111 households) of the total respondents assembled at the evacuation point immediately after the earthquake. Among those who assembled at the evacuation point, 43.2% were “very worried” and 51.4% were “worried” about the possibility of a tsunami (see Table 3). Compared with those who did not assemble at to the evacuation point, those who assembled at the evacuation point had smaller proportions of being slightly worried (2.7%) and not worried (2.7%) than those who did not assemble at the evacuation point. The chi-square test showed a statistically significant relationship between evacuation and worry about a tsunami.

Next, we investigate the relationship between worry about a tsunami and Natech damage estimation. Our survey asked the following question about Natech damage estimation, “Do you think that a tsunami (2–3 m high) would damage industrial facilities?” The simple tabulation demonstrates that 53.7% answered “Yes,” 16.9% responded “No,” and 29.4% selected “I don’t know.” We employed a detailed cross-tabulation to investigate the relationship between worry about the possibility of a tsunami and damage estimation caused by a tsunami by dividing the sample into two groups (those who assembled at the evacuation point and those who did not; see Table 4). In both groups, we find no statistically significant relationship between worry about the possibility of a tsunami and the estimation of the damage. The respondents of both groups selected “I don’t know [the damage estimation],” even if they were worried about a tsunami. These results imply that it was difficult for the respondents to imagine the cascading consequences at the time the earthquake occurred.

These chi-square tests do not verify the sequential steps of the response from evacuation to Natech damage estimation.

### 3.2. Risk Perception

As many relevant studies (e.g., (19,25,35)) suggest that risk perception is one of the significant predictors for people’s response, we analyzed whether our survey shows similar findings to the literature. We asked respondents whether they considered their village to be prone to earthquakes. The simple tabulation demonstrates that earthquake risk perception is not very high (see Table 4). The sum of “not prone” (24.9%) and “slightly prone” (42.8%) is bigger than that of “very prone” (8.7%) and “prone” (23.7%). Such a low perception indicates that people felt the earthquake occurred suddenly, and thus we can identify the earthquake in our case study as a sudden-onset disaster.

There is no statistical significance between the two groups (those who assembled at the evacuation point and those who did not) for earthquake risk perception. This indicates that the perceived earthquake risk does not have a significant relationship with evacuation behavior (see the upper part of Table 5). In addition, we find no significant relationship between risk perception and Natech damage estimation (see the middle part of Table 5). However, statistical significance is detected in the relationship between risk perception and worry about a tsunami (see the lower part of Table 5). Our results do not clearly show that risk perception has a significant impact on people’s responses.

### 3.3. Significant Variables Related to Natech Damage Estimation

We perform chi-square tests to identify significant variables listed in Table 1 relating to Natech damage estimation. As a result, access to the website of the National Disaster Management Agency (Badan Nasional Penanggulangan Bencana, or BNPB), access to the BMKG website, and online news access are significant (see Table 6). Village location has no significant relationship with Natech damage estimation (see Table 7). Regarding preparedness, we employ the following three types of preparedness: disaster risk reduction (DRR) activity participation; knowledge of the Indonesian Red Cross (Palang Merah Indonesia, PMI) activities; and knowledge of DRR-related groups and programs. None of the three preparedness variables have a significant relationship with Natech damage estimation (see Table 8).

Furthermore, we conducted a logistic regression analysis to examine whether other variables may be related to Natech damage estimation. The value of AIC in the model that adopted all of the 16 independent variables was 290.72, while the smallest value of AIC among the examined models was 275.56. As it is considered that the smaller value of AIC indicates a more suitable fit in terms of the statistical model, the authors determined the latter model as the best model. The best model showed that BNPB website access and online news access were significant at the 5% and 1% levels, respectively, while neither worry about the possibility of a tsunami nor evacuation behavior appeared to be related to the Natech damage estimation (see Table 9). The results indicate that the respondents were likely not to estimate Natech damages if they had accessed the BNPB website or online news. This result can be interpreted as follows. Information on Natech might not have been available on both the BNPB website and online news because a tsunami was not generated in the earthquake of our case study. Therefore, it is understandable that those who accessed the BNPB website or online news did not estimate Natech damage.

### 3.4. Village Location

The chi-square test reveals that there is no statistically significant difference between village location and people’s responses (see Table 10). The result for Natech damage estimation is shown in Table 7.

### 3.5. Information Access

Almost 80% (79.3%) of respondents accessed a TV news program after the earthquake, and this percentage was remarkably the highest (see Table 11). The chi-square test showed statistical significance for this behavior.

Except for TV news program and radio access, we find a statistical significance in each information source based on the chi-square tests. Those who assembled at the evacuation point tended to access information sources more and this pattern is quite clear.

It is notable that access to social media (24.3%) is greater than access to BNPB (17.1%) and BMKG (21.6%) websites. These results seem to reflect the current Indonesian context. During the interviews, we exemplified to the respondents that social media refers to Facebook, Twitter, WhatsApp, Line, Path, and Instagram. Although we need to pay attention to fake news (locally often described as “hoax problems”), social media enables people to observe what people close to them are doing and to gather unassessed information quickly.

Compared with the results of Yu et al. [19], information access in our survey was more active. In Yu et al. [19], 12% of the respondents tried to search for information. Their study dealt with the Great East Japan Earthquake and many affected areas faced a shortage of electricity supply. By contrast, there was no black out in our case study. We consider that this is the reason we have a big difference in information access between the two surveys. In addition to the findings of Feng et al. [29], people accessed information provided by the BNPB and BMKG as authority positions and got to know other people’s responses through social media.

Based on a hypothetical assumption that information may cause worries about the possibility of a tsunami, although chi-square tests are different to cause–effect analysis, three types of information access are found to be related to the worry: the BMKG website access, TV news program access, and social media access (see Table 12). Those who accessed these information sources tended to be less worried about the possibility of a tsunami. Regarding the relationship between information access and Natech damage estimation, we demonstrate the results in Table 6.

### 3.6. Preparedness

In the survey, we asked whether the respondents had ever participated in any type of drill/simulation/exercise/activities on DRR. About 30% respondents stated that they had (32.8%) (see Table 13a). However, such participation does not have a statistical relationship with evacuation immediately after an earthquake. We find no statistical significance between DRR activity participation and worry about the possibility of a tsunami (see Table 13b).

In addition, we clarify whether the respondents knew about DRR-related groups and programs. At the survey sites, we identified that the local actors, such as PMI, DRR Forum, Youth Group for Disaster Preparedness (Taruna Siaga Bencana, Tagana), Disaster Response Village (Desa Tanggap Bencana), Disaster Prepared Village (Kampung Siaga Bencana), Alert Village (Desa Siaga), and Sultan Ageng Tirtayasa University, had been implementing DDR activities. Among them, the PMI was the most active. When we consider the group of respondents who assembled at the evacuation point, 42.3% knew about PMI activities (see the left part of Table 14), which was a significantly higher proportion than those who did not assemble at the evacuation point. For the other six groups and programs, the results show the same tendency (see the right part of Table 14). Those who assembled at the evacuation point knew more about DRR activities. Regarding the relationship with worry about the possibility of a tsunami, awareness of PMI activities showed statistical significance (see the left part of Table 15).

Lindell et al. [25] addressed the effectiveness of pre-impact training activities, while Nakaya et al. [39] provided evidence to advocate the administration of tsunami drills in seaside communities to enhance evacuation behavior immediately after the disaster onset. Our study does not show such a strong implication. It is not easy to interpret why activity participation is not significant, but knowledge is. One possible interpretation is that local people witnessed advertising banners of relevant activities even though they did not attend them. For more detailed analysis for identifying effects of activity participation and knowledge, qualitative methods, such as in-depth interviews and focused group discussions, would be complementary to quantitative approaches.

Moreover, aspects of preparedness in the present study were limited, and further analysis is required. Theoretical frameworks such as the Social-Cognitive Model (SCM) [40,41] and the Protective Action Decision Model (PADM) [42] argued, in detail, a variety of factors influencing the adoption of preparedness. The SCM analyzed preparedness by setting a three-stage reasoning process (motivation to prepare, forming intentions to prepare, and their conversion into actual preparation). Paton et al. [41] clarified that the mechanism of “Intentions to Prepare” and “Intentions to Seek Information” are qualitatively different and stressed “This distinction has significant implications for conceptualising the preparedness process.” Findings from the PADM research support encouraging emergency preparedness during the continuing hazard phase (the time between incidents). These arguments enable the present study to extend a more general understanding of preparedness.

## 4. Conclusions

### 4.1. Summary

Immediately after the earthquake, almost 30% of the respondents assembled at the relevant evacuation point. We explored, in detail, whether their evacuation action was related to worries about a tsunami and Natech damage estimation. In addition, to compare the key findings provided by the relevant literature, we assessed differences in location, information access, perceived risk, and preparedness.

Based on the survey results, no sequential steps of people’s response were observed: evacuation right after the earthquake was related to worry about the possibility of a tsunami, but not to Natech damage estimation. The factors relevant to evacuation behavior were information access (except TV news program and radio), worry about the possibility of a tsunami, and knowledge of the DRR-related groups and programs. Meanwhile, the survey location (two villages), perceived earthquake risk, and DRR activity participation were less relevant to the behavior of assembling at the evacuation point. The survey results do not support the finding from the existing literature that higher risk perception is associated with evacuation behavior, nor do they support the assumption that a life-saving quick action is related to foreseeing cascading sequential consequences. At least based on our survey results, we consider that the importance of risk perception should not be excessively emphasized and needs to be further empirically evaluated.

### 4.2. Implications

In the earthquake that we used as a case study, shaking intensity was not so strong, and people did not face building collapse, at least in Cilegon. In addition, the earthquake occurred during the day. In this situation, people were able to access several information sources and seemed to be able to decide whether to take actions or not. However, at the same time, they needed to process and digest a lot of information, and they were likely to have been confused because few people anticipated the earthquake (see the upper part of Table 5). Although further clarification is necessary, some information raised or mentioned concerns about a tsunami, which is why people were worried about it. The results of Natech damage estimation imply that the content of information provided after disasters needs to be improved to easily make people understand that Natech situations need to be considered.

Some studies suggested to utilize the critical moment right after the hazard event occurrence for science communication and education campaigns [43,44]. The affected people urgently look for vital information as long as the communication line works, while families and friends of the potentially affected people also urgently search relevant information. For those who do not have anyone to care for in the affected zones, it seems very effective to provide learning opportunities. Specifically considering the country contexts in Indonesia, there have been growing concerns about the fake news problems [45,46]. Possibilities of large-scale information dissemination after the incidents should be carefully considered.

Our key finding is that sequential steps of people’s response are not easily organized. However, it solely relied on one local study. For further verification, more studies are needed. There is a port area in Sendai city (Miyagi Prefecture, Japan), and the characteristics of the port area are similar to the case of the present study: the port area has some industrial facilities, and the residential area is located near the port. In the Great East Japan Earthquake (GEJE), the area experienced the Natech after the strong shaking and tsunami arrival [19]. After GEJE, at least, the Sendai Port area experienced two relatively bigger earthquakes. The first earthquake occurred in November 2016. Another earthquake happened in February 2021. While the epicenters of these two earthquakes were both located off Fukushima Prefecture, relatively strong shaking was observed around the Sendai Port area. We assume that higher risk perception and worries about a tsunami would be expected in the Sendai Port area based on its own experience. Preparedness might be a key factor for determining the Natech damage estimation, since a variety of practices have been carried out in that area.

### 4.3. Limitations

Our analysis was mainly limited to simple tabulations and statistical analysis using the chi-square test, except one trial of logistic regression analysis. In addition, we explored only the relationship between variables and did not analyze multiple variables concurrently (e.g., multi-variable analysis techniques). The reason that we did not conduct multi-variable analysis is that some answers to our questions are binary and categorical, and they are not suitable for multi-variable analysis. For example, when asking about information seeking, we simply asked if the respondents accessed the government agencies (Yes/No style). To test our initial hypothetical assumption, structural equation modeling and path analysis would be more suitable. The use of such approaches is essential to advance research in this area. Furthermore, our household survey made it difficult to include individual demographics in the analysis.

## Figures and Tables

**Table 1:** Classification of variables influencing behavior.

Relevant Variables from the Existing Literature	Variables in the Present Study
Risk perception	Proneness of risk as perceived risk
Emotion	Worry about the possibility of a tsunami
Direction	Village location
Impact estimation	Natech damage estimation
Information seeking Reference to authority positions	Information access, including social media and government agencies
Preparedness	Disaster risk reduction activity participation, knowledge

**Table 2:** Average of net wage/salary per month of formal employee by province and main occupation (Indonesia rupiahs).

	Year 2018	Year 2020
Banten Province	3,468,768	3,693,411
Jakarta Special Province	4,523,453	4,224,720
Average by Province	3,592,501	2,756,345

Source: BPS-Statistics Indonesia [37,38]. Note: As our survey was implemented in 2018, we introduce data of Year 2018. Data of Year 2020 are the latest information, but the value seems to be influenced by COVID-19.

**Table 3:** Worry about the possibility of a tsunami.

		Worry about the Possibility of a Tsunami			
		Very Worried	Worried	Slightly Worried	Not Worried
Assembled at evacuation point (n = 111)		43.2%	51.4%	2.7%	2.7%
No (n = 267)		56.6%	35.6%	4.5%	3.4%
Sum (N = 378)		52.6%	40.2%	4.0%	3.2%
χ^2^ (3, N = 378) = 8.233, *p* < 0.05.					

**Table 4:** Natech damage estimation.

			Were You Worried about Whether the Earthquake Would Generate a Tsunami?			
			Very Worried	Worried	Slightly Worried	Not Worried
Assembled at evacuation point	Do you think that a tsunami (2–3 metres in height) would cause damage?	Yes (n = 59)	42.4%	54.2%	1.7%	1.7%
		No (n = 23)	47.8%	39.1%	4.3%	8.7%
		I don’t know. (n = 28)	39.3%	57.1%	3.6%	0.0%
	Sum (N = 110)		42.7%	51.8%	2.7%	2.7%
*χ^2^* (6, N = 110) = 5.677, *n.s.*						
No (did not assemble at evacuation point)	Do you think that a tsunami (2–3 metres in height) would cause damage?	Yes (n = 144)	59.0%	33.3%	3.5%	4.2%
		No (n = 41)	70.7%	26.8%	0.0%	2.4%
		I don’t know. (n = 81)	44.4%	44.4%	8.6%	2.5%
	Sum (N = 266)		56.4%	35.7%	4.5%	3.4%
*χ^2^* (6, N = 266) = 12.413, *n.s.*						
Total	Do you think that a tsunami (2–3 metres in height) would cause damage?	Yes (n = 203)	54.2%	39.4%	3.0%	3.4%
		No (n = 64)	62.5%	31.3%	1.6%	4.7%
		I don’t know. (n = 109)	43.1%	47.7%	7.3%	1.8%
	Sum (N =376)		52.4%	40.4%	4.0%	3.2%
*χ^2^* (6, N = 376) = 11.650, *n.s*						

**Table 5:** Relationship between perceived earthquake risk and people’s response.

	How Prone Is Your Village to an earthquake?			
	Very Prone	Prone	Slightly Prone	Not Prone
Assembled at evacuation point (n = 47)	8.5%	17.0%	48.9%	25.5%
No (n = 126)	8.7%	26.2%	40.5%	24.6%
Sum (N = 173)	8.7%	23.7%	42.8%	24.9%
*χ^2^* (3, N = 173) = 1.801, *n.s.*				
	How prone is your village to an earthquake?			
	Very prone	Prone	Slightly prone	Not prone
Natech damage estimation—Yes (n = 103)	10.7%	25.2%	35.9%	28.2%
Natech damage estimation—No (n = 20)	15.0%	25.0%	35.0%	25.0%
Natech damage estimation—I don’t know (n = 50)	2.0%	20.0%	60.0%	18.0%
Sum (N = 173)	8.7%	23.7%	42.8%	24.9%
*χ^2^* (6, N = 173) = 10.654, *n.s.*				
Worry about the possibility of a tsunami	How prone is your village to an earthquake?			
	Very prone	Prone	Slightly prone	Not prone
Very worried. (n = 84)	10.7%	35.7%	41.7%	11.9%
Worried (n = 74)	8.1%	10.8%	41.9%	39.2%
Slightly worried (n = 8)	0.0%	37.5%	37.5%	25.0%
Not worried (n = 7)	0.0%	0.0%	71.4%	28.6%
Sum (N = 173)	8.7%	23.7%	42.8%	24.9%
*χ^2^* (9, N = 173) = 27.5945 *p* < 0.01				

**Table 6:** Natech damage estimation and information access (N = 378).

	1. BNPB *^1^ Website Accessed	No	2. BMKG *^2^ Website Accessed	No
Natech damage estimation—Yes (n = 203)	7.4%	92.6%	7.4%	92.6%
Natech damage estimation—No (n = 64)	20.3%	79.7%	20.3%	79.7%
Natech damage estimation—I don’t know (n = 111)	8.1%	91.9%	8.1%	91.9%
	*χ^2^* (2, N = 378) = 9.706, *p* < 0.01		*χ^2^* (2, N = 378) = 9.706 *p* < 0.01	
	3. TV news program accessed	No	4. Radio accessed	No
Natech damage estimation—Yes (n = 203)	78.3%	21.7%	0.5%	99.5%
Natech damage estimation—No (n = 64)	78.1%	21.9%	1.6%	98.4%
Natech damage estimation—I don’t know (n = 111)	82.0%	18.0%	1.8%	98.2%
	*χ^2^* (2, N = 378) = 0.658, *n.s*		*χ^2^* (2, N = 378) = 1.362, *n.s*	
	5. Online news accessed	No	6. Social media*^3^ accessed	No
Natech damage estimation—Yes (n = 203)	4.4%	95.6%	14.3%	85.7%
Natech damage estimation—No (N = 64)	20.3%	79.7%	21.9%	78.1%
Natech damage estimation—I don’t know (n = 111)	0.9%	99.1%	20.7%	79.3%
	*χ^2^* (2, N = 378) = 28.860, *p* < 0.01		*χ^2^* (2, N = 378) = 3.104, *n.s*	

*1 Badan Nasional Penanggulangan Bencana (National Disaster Management Agency in English) *2 Badan Meteorologi Klimatologi dan Geofisika (Meteorological, Climatological, and Geophysical Agency in English) *3 In the survey, the authors explained to the respondents that social media means Facebook, Twitter, WhatsApp, Line, Path, and Instagram.

**Table 7:** Natech damage estimation and village location (N = 378).

	Lebak Gede (n = 230)	Gunung Sugih (n = 148)
Natech damage estimation—Yes (n = 203)	58.1%	41.9%
Natech damage estimation—No (n = 64)	73.4%	26.6%
Natech damage estimation—I don’t know (n = 111)	58.6%	41.4%
*χ^2^* (2, N = 378) = 0.116, *n.s*		

**Table 8:** Natech damage estimation and preparedness (N = 378).

	DRR*^1^ Activities Participation—Yes	No
Natech damage estimation—Yes (n = 202)	35.1%	64.9%
Natech damage estimation—No (n = 63)	27.0%	73.0%
Natech damage estimation—I don’t know (n = 111)	31.5%	68.5%
	*χ^2^* (2, N = 376) = 1.554, *n.s*	
	PMI*^2^ activities—Known	No
Natech damage estimation—Yes (n = 202)	31.0%	69.0%
Natech damage estimation—No (n = 63)	40.6%	59.4%
Natech damage estimation—I don’t know (n = 111)	28.8%	71.2%
	*χ^2^* (2, N = 378) = 2.787, *n.s*	
	DRR-related groups and programs—Known	No
Natech damage estimation—Yes (n = 202)	21.2%	78.8%
Natech damage estimation—No (n = 63)	34.4%	65.6%
Natech damage estimation—I don’t know (n = 111)	26.1%	73.9%
	*χ^2^* (2, N = 378) = 4.666, *n.s*	

*1 Disaster Risk Reduction *2 Palang Merah Indonesia (Indonesian Red Cross in English).

**Table 9:** Results of logistic regression analysis.

	*B*	(SE)
(Constant)	1.493 **	(0.1710)
BNPB website access	−1.304 *	(0.5478)
Online news access	−1.492 **	(0.4903)
PMI activity [Risk mapping with local community participation]	−1.079 ^†^	(0.6482)
*N*	265	
Akaike Information Criterion	275.56	
Nagelkerke *R^2^*	0.125	

** *p* < 0.01, * *p* < 0.05, ^†^ *p* < 0.1.

**Table 10:** Evacuation behavior by village location.

	Lebak Gede (n = 231)	Gunung Sugih (n = 149)
Assembled at evacuation point (n = 111)	59.5%	40.5%
No (n = 269)	61.3%	38.7%
*χ^2^* (1, N = 380) = 0.116, *n.s*		
	Lebak Gede (n = 230)	Gunung Sugih (n = 148)
Worry about the possibility of a tsunami—Very worried (n = 199)	62.8%	37.2%
Worried (n = 152)	55.3%	44.7%
Slightly worried (n = 15)	80.0%	20.0%
Not worried (n = 12)	75.0%	25.0%
*χ^2^* (3, N = 378) = 5.631, *n.s*		

**Table 11:** Information access after the earthquake (N = 380).

1. BNPB *^1^ Website	Accessed	No
Assembled at evacuation point (n = 111)	17.1%	82.9%
No (n = 269)	1.1%	98.9%
*χ^2^* (1, N = 380) = 36.889, *p* < 0.01		
2. BMKG^*2^ website	Accessed	No
Assembled at evacuation point (n = 111)	21.6%	78.4%
No (n = 269)	4.8%	95.2%
*χ^2^* (1, N = 380) = 25.200, *p* < 0.01		
3. TV news programs	Accessed	No
Assembled at evacuation point (n = 111)	79.3%	20.7%
No (n = 269)	79.2%	20.8%
*χ^2^* (1, N = 380) = 0.000, *n.s*		
4. Radio	Accessed	No
Assembled at evacuation point (n = 111)	2.7%	97.3%
No (n = 269)	0.4%	99.6%
*χ^2^* (1, N = 380) = 4.099, *n.s*		
5. Online news	Accessed	No
Assembled at evacuation point (n = 111)	11.7%	88.3%
No (n = 269)	3.7%	96.3%
*χ^2^* (1, N = 380) = 8.831, *p* < 0.01		
6. Social media*^3^	Accessed	No
Assembled at evacuation point (n = 111)	24.3%	75.7%
No (n = 269)	14.9%	85.1%
*χ^2^* (1, N = 380) = 4.836, *p* < 0.05		

*1 Badan Nasional Penanggulangan Bencana (National Disaster Management Agency in English) *2 Badan Meteorologi Klimatologi dan Geofisika (Meteorological, Climatological, and Geophysical Agency in English) *3 In the survey, the authors explained to the respondents that social media means Facebook, Twitter, WhatsApp, Line, Path, and Instagram.

**Table 12:** Information access and worry about the possibility of a tsunami (N = 378).

Worry about the Possibility of a Tsunami	1. BNPB*^1^ Website Accessed	No	2. BMKG*^2^ Website Accessed	No
Very worried (n = 199)	4.5%	95.5%	6.5%	93.5%
Worried (n = 152)	7.2%	92.8%	10.5%	89.5%
Slightly worried (n = 15)	13.3%	86.7%	26.7%	73.3%
Not worried (n = 12)	0.0%	100.0%	33.3%	66.7%
	*χ^2^* (3, N = 378) = 3.454, *n.s*		*χ^2^* (3, N = 378) = 14.855, *p* < 0.01	
Worry about the possibility of a tsunami	3. TV news program accessed	No	4. Radio accessed	No
Very worried (n = 199)	78.9%	21.1%	1.0%	99.0%
Worried (n = 152)	83.6%	16.4%	1.3%	98.7%
Slightly worried (n = 15)	60.0%	40.0%	0.0%	100.0%
Not worried (n = 12)	58.3%	41.7%	0.0%	100.0%
	*χ^2^* (3, N = 378) = 8.330, *p* < 0.05		*χ^2^* (3, N = 378) = 0.390, *n.s*	
Worry about the possibility of a tsunami	5. Online news accessed	No	6. Social media*^3^ accessed	No
Very worried (n = 199)	7.5%	92.5%	19.1%	80.9%
Worried (n = 152)	3.9%	96.1%	12.5%	87.5%
Slightly worried (n = 15)	6.7%	93.3%	53.3%	46.7%
Not worried (n = 12)	8.3%	91.7%	16.7%	83.3%
	*χ^2^* (3, N = 378) = 2.065, *n.s*		*χ^2^* (3, N = 378) = 16.153, *p* < 0.01	

*1 Badan Nasional Penanggulangan Bencana (National Disaster Management Agency in English) *2 Badan Meteorologi Klimatologi dan Geofisika (Meteorological, Climatological, and Geophysical Agency in English) *3 In the survey, the authors explained to the respondents that social media means Facebook, Twitter, WhatsApp, Line, Path, and Instagram.

**Table 13:** Preparedness (disaster risk reduction (DRR) activity participation) and (a) immediate evacuation (N = 378) and (b) worry about the possibility of a tsunami (N = 376).

(a) Immediate evacuation	Yes	No
Assembled at evacuation point (n = 110)	40.0%	60.0%
No (n = 268)	29.9%	70.1%
*χ^2^* (1, N = 378) = 3.644, *n.s*		
(b) Worry about the possibility of a tsunami	Yes	No
Very worried (n = 198)	31.8%	68.2%
Worried (n = 151)	35.1%	64.9%
Slightly worried (n = 15)	40.0%	60.0%
Not worried (n = 12)	16.7%	83.3%
*χ^2^* (3, N = 376) = 2.207, *n.s*		

**Table 14:** Preparedness (knowledge) and immediate evacuation (N = 380).

	PMI*^1^ Activities		DRR*^2^-Related Groups and Programs	
	**Known**	**No**	**Known**	**No**
Assembled at evacuation point (n = 111)	42.3%	57.7%	37.8%	62.2%
No (n = 269)	27.9%	72.1%	19.7%	80.3%
*χ^2^* (1, N = 380) = 7.539, *p* < 0.05			*χ^2^* (1, N = 380) = 13.783, *p* < 0.01	

*1 Palang Merah Indonesia (Indonesian Red Cross in English) *2 Disaster Risk Reduction.

**Table 15:** Preparedness (knowledge) and worry about the possibility of a tsunami (N = 378).

	PMI*^1^ Activities		DRR*^2^-Related Groups and Programs	
Worry about the Possibility of a Tsunami	Known	No	Known	No
Very worried (n = 199)	32.7%	67.3%	21.6%	78.4%
Worried (n = 152)	31.6%	68.4%	28.3%	71.7%
Slightly worried (n = 15)	53.3%	46.7%	46.7%	53.3%
Not worried (n = 12)	0.0%	100.0%	8.3%	91.7%
*χ^2^* (3, N = 378) = 8.835, *p* < 0.05			*χ^2^* (3, N = 378) = 7.655, *n.s*	

*1 Palang Merah Indonesia (Indonesian Red Cross in English) *2 Disaster Risk Reduction.

## Data Availability

The data presented in this study are available on request from the corresponding author.
